# Valorisation of corncob into furfuryl alcohol and furoic acid via chemoenzymatic cascade catalysis

**DOI:** 10.1186/s40643-021-00466-3

**Published:** 2021-11-16

**Authors:** Jiacheng Ni, Junhua Di, Cuiluan Ma, Yu-Cai He

**Affiliations:** 1 National-Local Joint Engineering Research Center of Biomass Refining and High-Quality Utilization, School of Pharmacy, Changzhou University, Changzhou, China; 2 State Key Laboratory of Biocatalysis and Enzyme Engineering, Hubei Collaborative Innovation Center for Green Transformation of Bio-resources, Hubei Key Laboratory of Industrial Biotechnology, School of Life Sciences, Hubei University, Wuhan, China

**Keywords:** Furans, Sn-GP, Biomass, Co-catalysis, Chemoenzymatic catalysis

## Abstract

Heterogeneous tin-based sulfonated graphite (Sn-GP) catalyst was prepared with graphite as carrier. The physicochemical properties of Sn-GP were captured by FT-IR, XRD, SEM and BET. Organic acids with different pKa values were used to assist Sn-GP for transforming corncob (CC), and a linear equation (Furfural yield  = − 7.563 ×  pKa  + 64.383) (*R*^2^  =  0.9348) was fitted in acidic condition. Using sugarcane bagasse, reed leaf, chestnut shell, sunflower stalk and CC as feedstocks, co-catalysis of CC (75.0 g/L) with maleic acid (pKa  =  1.92) (0.5 wt%) and Sn-GP (3.6 wt%) yielded the highest furfural yield (47.3%) for 0.5 h at 170 °C. An effective furfural synthesis was conducted via co-catalysis with Sn-GP and maleic acid. Subsequently, *E. coli* CG-19 and TS completely catalyzed the conversion of corncob-derived FAL to furfurylalcohol and furoic acid, respectively. Valorisation of available renewable biomass to furans was successfully developed in tandem chemoenzymatic reaction.

**Graphical Abstract:**

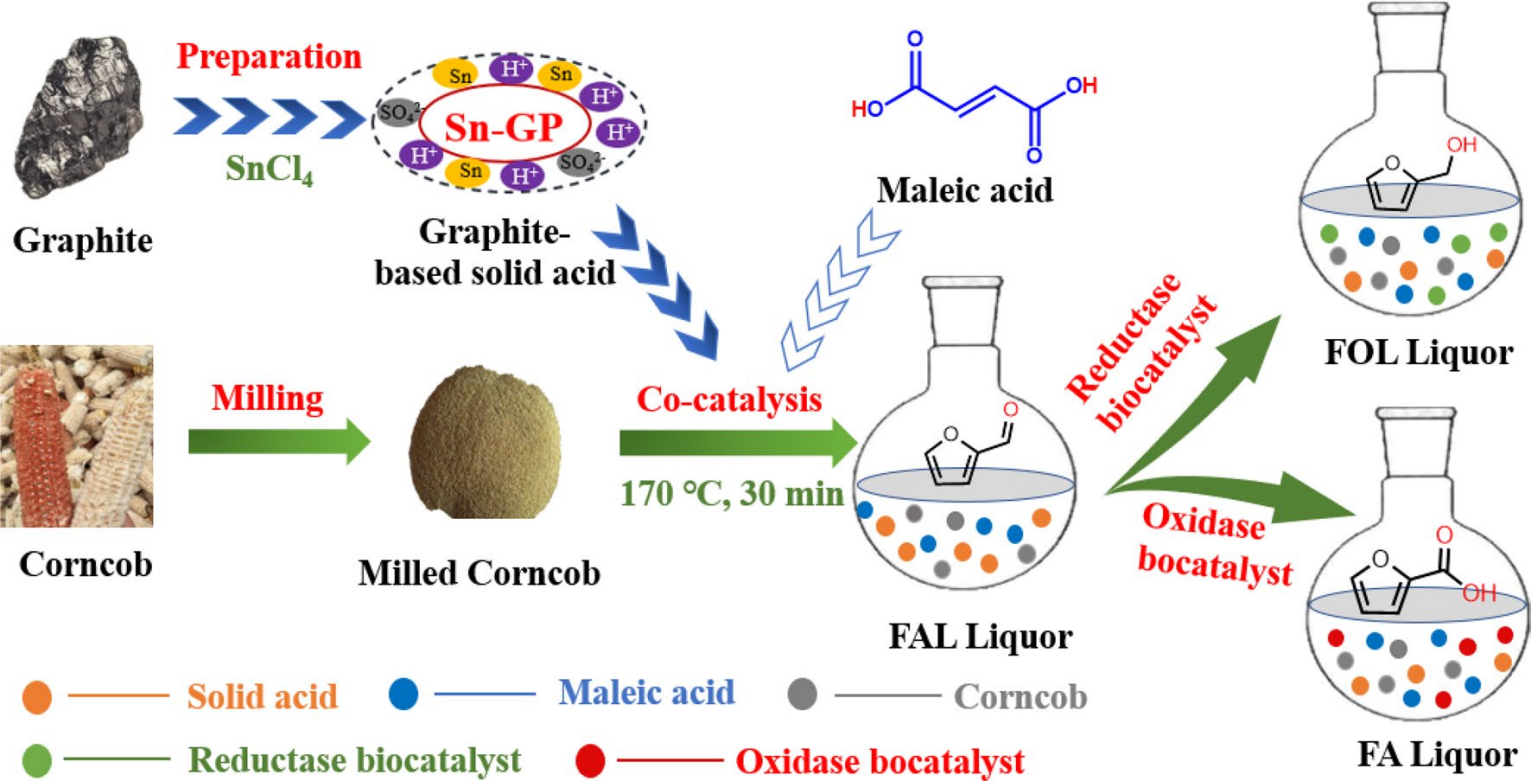

**Supplementary Information:**

The online version contains supplementary material available at 10.1186/s40643-021-00466-3.

## Introduction

In the past few decades, the rapid depletion of fossil fuels and serious environmental pollution have arouse to discover sustainable resources to replace non-renewable fossil resources. Biomass is attracting more and more attention as the only carbon-containing renewable energy source (Mohammadi Moradian et al. 2021; Narisetty et al. 2021; Yang et al. 2017). Lignocellulose (LB), a major category of biomass, is composed of three major components: lignin, cellulose and hemicellulose, and it has been used to efficiently manufacture a series of important biofuels and bio-based chemicals (Wang et al. 2017; Jin et al. 2016; Xia et al. 2020). Hemicelluloses is a major component of lignocellulose. It is consisted of β-(1,4)-glycosidic bonds linking C_5_ and C_6_ sugars in a multiphase polymer (Janker-Obermeier et al. 2012; Perkins et al. 2018). Xylan is an important polysaccharide existing in hemicelluloses of lignocellulosic biomass, which is further converted to produce *D*-xylose in acidic condition, and then dehydrated to produce furfural (FAL) (Wang et al. 2019a, b). FAL, as one of the ten most valuable bio-based building materials highlighted by the United States Department of Energy, has a wide range of applications in many industries, such as medicine, agriculture, chemicals, and cosmetics (Widsten et al. 2018; Yang et al. 2021). FAL is also utilized in the production of furans, such as furfuralcohol (FOL), furoic acid (FA), etc., (Cai et al. 2014; Cui et al. 2019; Weingarten et al. 2010; Zhang et al. 2020b).

Recently, many efforts have been made for production of furans (e.g., FOL and FA) via biocatalysis in environmentally friendly approaches (Choudhary et al. 2016). FOL is an important furan-biobased chemical with a wide range of applications in the agricultural, foundry, fuel, synthetic fiber and rubber industries (Li et al. 2021a, b). Bioreduction of FAL is an alternative route for FOL production. *S. cerevisiae* transformed FAL (62 mM) to FOL in 87% yield (Yan et al. 2019). *B. coagulans* reduced FAL to FOL in 87% yield in water-dioctyl phthalate (Bu et al. 2020). *M. deltae* converted low loading of FAL (10 mM) to FOL in 100% yield (Li et al. 2017). Furoic acid (FA) is also a key furan biobased chemical, which can be obtained by oxidation of FAL (Shi et al. 2019). It has been widely used in the production of flavors, polymers, fragrances, agrochemicals and pharmaceuticals (Kambara et al. 2010). FAL was oxidized to FA using *B. cereus* at 30 °C with a yield of 95% (Mitsukura et al. 2004). Immobilized short yellow *Bacillus* cells completely oxidized FAL to FA at 30 °C for 24 h (Ma et al. 2020). It is necessary to develop sustainable processes for transforming biomass to FOL and FA via chemoenzymatic cascade catalysis. One-pot FOL production via dehydration of corncob-derived xylose into FAL (51.9 mM) at 44% yield by SO_4_^2−^/SnO_2_-attapulgite (3.6 wt%) at 170 °C for 20 min followed by bioreduction of xylose-derived FAL into FOL at 100 yield with *E. coli* CCZU-A13 cells at 30 °C and pH 6.5 for 8 h (He et al. 2017a). One-pot FOL production via dehydration of corncob-derived xylose into FAL (70.2 mM) by SO_4_^2−^/SnO_2_-attapulgite (3.6 wt%) at pH 1.0 and 170 °C for 30 min followed by oxidation of AP-BSS-derived FAL (30 mM) into FA at 100% yield with *E. coli* BH cells at 35 °C and pH 7.5 for 200 h (Yang et al. 2020). However, biomass was required to pretreat prior to FAL synthesis, and the FAL loading was too low for the biological valorisation. Thus, effective conversion of biomass into FAL with high activity and biocompatible chemocatalyst and transformation of biomass-derived FAL into FOL and FA with robust biocatalysts need be developed.

To effectively synthesize FOL and FA using biomass-derive FAL as substrate, enhanced FAL yield from lignocellulosic biomass is attracted much attentions (Li et al. 2021a, b). Various homogeneous acids are widely utilized to transform hemicellulose into FAL. Compared to inorganic acids, organic acids are relatively less corrosive, but not as efficient in catalysis (Rong et al. 2012; Yang et al. 2012). Using HCOOH as catalyst, oil palm could be converted into FAL in 36% yield for 20 min at 280 °C (Tau et al. 2016). Compare to homogeneous inorganic acids, heterogeneous catalysts have been widely used in organic reactions as a class of green catalysts (Cao et al. 2020). Very recently, much attentions have been attracted to heterogeneous catalysts for FAL production due to their high stability, low corrosion, high catalytic activity, good recyclability (Gupta and Paul 2014; Li et al. 2019a, b, c). Numerous heterogeneous catalysts, such as mesoporous molecular sieve MCM-41 (Garcia-Sancho et al. 2013), metal oxides (Kaiprommarat et al. 2016), zeolites (Metkar et al. 2015), and resins (Sádaba Ojeda et al. 2014), have been utilized to convert biomass or *D*-xylose into FAL. Very recently, graphite (GP), an isomer of carbon, is chemically stable and corrosion resistant. It is a good raw material for synthesizing solid acids, which has attracted a lot of attention (Sajadi et al. 2021). GP is generally accepted to be a biocompatible material (Pankratov et al. 2016). GP-based carbon-nitride heterogeneous catalyst was prepared for dihydropyrimidinone synthesis (Ali et al. 2019). Graphite oxide (GO) was used as catalyst to open epoxides for the preparation of β-alkoxy alcohols (Maryam et al. 2014). The transformation of alcohols to amides in 69–95% yields was catalyzed using GO at 50 °C (Mirza-Aghayan et al. 2016). To enhance FAL yield, the effective catalytic process might be attempted to catalyze biomass using GP-based solid acid as catalyst.

Tin-based solid acid could be used for efficiently transforming biomass to FAL (Teng et al. 2020). Acidified heterogeneous Sn-sepiolite (3.0 wt%) transformed alkali-pretreated dewaxed rice straw into furfural at 42% yield at 170 °C for 20 min (Peng et al. 2019). 90.3 mM furfural was obtained from the alkali pretreatment of dewaxed corncob (75 g/L) at 170 °C for 0.5 h with acidified Sn-ZRD catalyst (3.6 wt%, pH 1.0) in the aqueous media (Zhang et al. 2019). GP is generally accepted to be a biocompatible material (Pankratov et al. 2016). In this work, heterogeneous catalyst Sn-GP was synthesized using graphite (GP) as support, and the structure properties of Sn-GP were captured by FT-IR, XRD, SEM and BET. To enhance the FAL yield from biomass, co-catalysis of Sn-GP with dilute organic acids was attempted to synthesize FAL. Various reaction factors were examined on FAL yields. Using sugarcane bagasse, reed leaf, chestnut shell, sunflower stalk and CC as feedstocks, co-catalysis of biomass into FAL was demonstrated. Subsequently, six biocatalyts were used to transform biomass-derived FAL to synthesize furans (FOL and FA). Sustainable transformation of available, inexpensive, abundant, and renewable biomass to furans was successfully developed via one-pot chemoenzymatic cascade catalytic reaction.

## Materials and methods

### Materials

Sugarcane bagasse (SCB) obtained the suburb of Guilin (Guangxi Province, China). Reed leaf (RL), chestnut shell (CNS), sunflower stalk (SFS) and corncob (CC) was obtained the suburbs of LuAn (Anhui Province, China). Sodium hydroxide (NaOH), ammonia, SnCl_4_·5H_2_O, sulfuric acid (H_2_SO_4_), β-*D*-1-thiogalactopyranoside (IPTG), maleic acid, fructose, sucrose, cellobiose, mannose, glucose, NAD^+^, *D-*xylose, ampicillin and other reagents were bought from Whanhan Macklin Co., Ltd., (Wuhan, P.R. China).

### Preparation of Sn-GP catalyst using GP as carrier

A turbid solution was obtained by blending SnCl_4_ 5H_2_O (40.0 g), GP (84.0 g) and ethanol (1.5 L) at room temperature, and then ammonia (25 wt%) was dripped into this solution until neutral. The generated colloidal mixture was tandemly oven-dried at 70 °C (15 h) and 90 °C (15 h). The dried solid powder was mixed with 120.0 mL H_2_SO_4_ (0.50 M) for 180 min. After filtration, sulfonated powders were dried in an oven (90 °C, 12 h) and calcinated at 550 °C for 240 min. The sulfonated Sn-GP catalyst was used to catalyze the CC to FAL.

### Co-catalysis of CC into FAL with Sn-GP and organic acids

To improve the FAL yield, various organic acids (maleic acid, fumaric acid, oxalic acid, glyoxalic acid, malic acid, citric acid, formic acid, succinic acid, and propionic acid) (0.5 wt%) were added separately to a 40 mL water containing CC (75.0 g/L, 40–60 mesh) and Sn-GP (1.44 g, 3.6 wt%) in a reactor (170 °C) for 0.5 h. To examine the dosage of maleic acid on FAL production in a 170 °C reactor, the mixture of maleic acid (0.1–0.9 wt%) and Sn-GP (1.44 g, 3.6 wt%) was used for co-catalysis of CC (75 g/L) in 40 mL water for 0.5 h. To evaluate SO_4_^2−^/SnO_2_-GP dose, catalytic time and temperatures on the influence of transforming CC into FAL in 100-mL reactor (500 rpm), CC (75 g/L) was mixed with water (40 mL), Sn-GP (0–6.0 wt%) and maleic acid (0.35 wt%) at 140–180 °C for 10–50 min. The FAL yield was calculated as follows:

1 $$ {\text{FAL}}\;{\text{yield}}\;(\% ) = \frac{{{\text{FAL}}\;{\text{produced}}\;({\text{g}}) \times 0.88}}{{{\text{Xylan}}\;{\text{in}}\;{\text{biomass}}}} \times \frac{150}{{96}} \times 100 $$

where 150 and 96 represent molecular weight (g/mol) of *D*-xylose and FAL, respectively. When raw biomass was used as feedstock, the FAL yield was calculated by forming *D*-xylose content to equivalent amount of xyan in biomass by times a conversion factor of 0.88.

### Biotransformation of FAL into FOL or FA

Three recombinant *E. coli* strains harboring FAL-reducing activity (*E. coli* CG-19, *E. coli* K14, and *E. coli* A13) and three recombinant *E. coli* strains harboring dehydrogenase activity (*E. coli* TS, *E. coli* HMFOMUT, and *E. coli* BH) were activated for 8 h at 30 °C on Luria–Bertani (LB) medium supplemented kanamycin (50 mg/L). The cells were then cultured in Terrific Broth medium (He et al. 2017a) containing kanamycin (50 mg/L) (or ampicillin) at 30 °C until cells grew to an OD_600_ of 0.60, then isopropyl β-*D*-1-thiogalactopyranoside (IPTG) was supplemented to Terrific Broth medium and cells were incubated for 14–16 h on shaking (25 °C). Finally, cells were recovered by washing with NaCl (0.75%) and centrifugation (8000×*g*, 8 min) and stored in a refrigerator at 4 °C (Li et al. 2021a, b). CC (7.5 wt%), Sn-GP (3.6 wt%), maleic acid (0.5 wt%) and distilled water (40 mL) were mixed in a 100-mL reactor. CC After 30 min of catalysis in this medium (pH 1.0) at 170 °C and 500 rpm, the solution’s pH was regulated to suitable pH for biotransformation. FAL was biologically reduced to FOL with *E. coli* CG-19, *E. coli* K14, or *E. coli* A13 wet cells (50.0 g/L) by supplementary of co-substrate glucose (glucose-to-FAL molar ratio 1.5:1) at pH 7.5 and 35 °C. FAL was oxidized to FA with *E. coli* TS, *E. coli* HMFOMUT, or *E. coli* BH wet cells (50.0 g/L) at pH 7.0 and 30 °C. The FOL yield and FA yield, were calculated as follows:

2 $$ {\text{FOL}}\;{\text{yield}}\;(\% ) = \frac{{{\text{FOL}}\;{\text{produced}}\;{\text{(mM)}}}}{{{\text{Initial}}\;{\text{FAL}}\;{\text{(mM)}}}} \times 100 $$

3 $$ {\text{FA}}\;{\text{yield}}\;(\% ) = \frac{{{\text{FA}}\;{\text{produced}}\;{\text{(mM)}}}}{{{\text{Initial}}\;{\text{FAL}}\;{\text{(mM)}}}} \times 100 $$

### Analytical methods

X-ray diffraction (XRD), scanning electron microscopy (SEM), Fourier transform infrared spectroscopy (FT-IR), and Brunauer–Emmett–Teller (BET) were used for the characterization of Sn-GP. Biomass components were analyzed as reported NREL procedure. Glucose, *D*-xylose, and FAL were measured by HPLC, and FOL was determined by GC.

## Results and discussion

### Characterization of Sn-GP

**Fig. 1:**
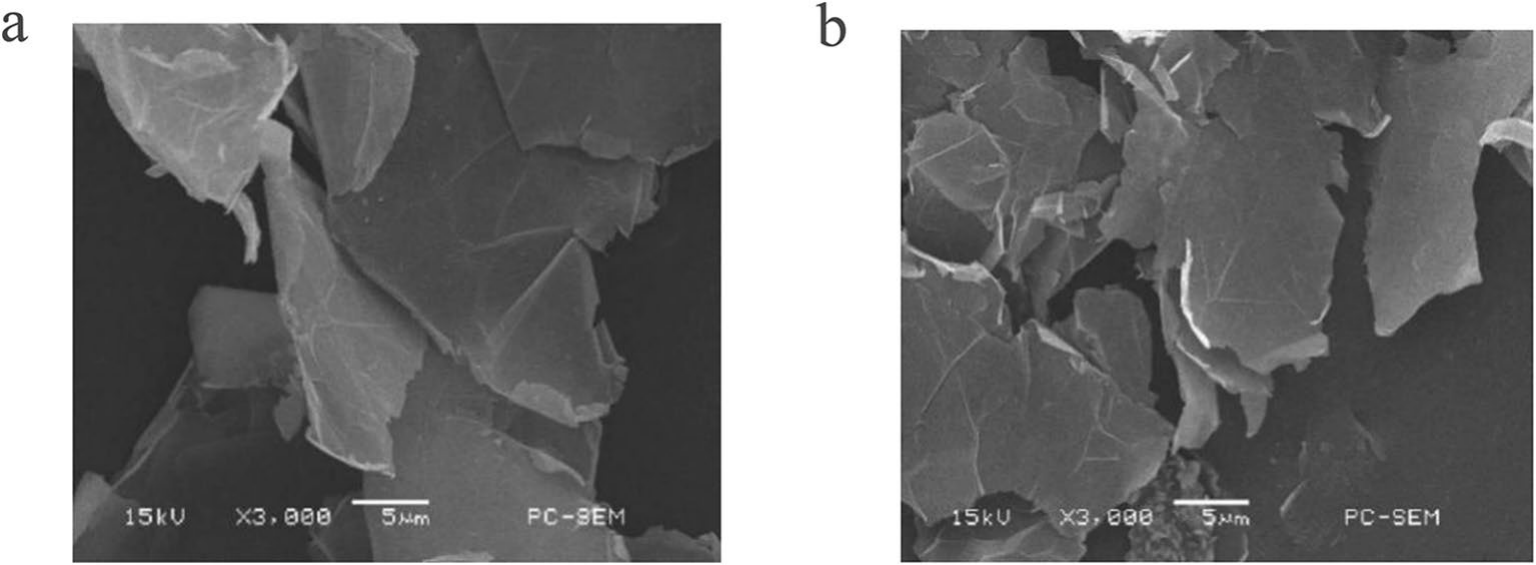
SEM image of GP (**a**) and Sn-GP (**b**)

Sulfonated GP catalysts loaded with tin were prepared using GP as carrier, and their surface and pore properties were determined by BET, SEM, XRD and FT-IR. The surface and pore change of Sn-GP was measured by BET method (Additional file 1: Table S1). Relative to the carrier GP, the specific surface area (SSA) of Sn-GP increased from 0.5 to 29.4 m^2^/g, the pore volume (PV) expanded from 0.01 to 0.03 cm^3^/g, and the pore diameter (PD) reduced from 135.2 to 5.8 nm. The increased SSA and PV might be ascribed to the action of solvents, SO_4_^2−^ and Sn^4+^ during the preparation of Sn-GP. The embedding of Sn^4+^ and SO_4_^2−^ into GP layers could influence the pore structure of GP (Chen et al. 2021). Probably, some micropores in GP might be blocked by Sn^4+^. Compared with fresh GP, Sn-GP had the rougher surface. As revealed in SEM (Fig. 1a, b), the Sn-GP surface was mostly fractured and rougher than that of GP. The rough and porous structure might facilitate the loading of Sn^4+^ and SO_4_^2−^ and provide the contact between the heterogeneous catalyst surface and the substrate (Li et al. 2020). The FT-IR analysis of GP and Sn-GP was conducted (Additional file 1: Fig. S1A). The peak near 1630 cm^−1^ was related to OH stretching and bending vibrations for H_2_O adsorbed on GP surface. The peak near 1400 cm^−1^ was attributed to the absorption peak by the contraction vibration of the tertiary hydroxyl group. The peaks about 1030 and 1140 cm^−1^ were attributed to S  =  O (Li et al. 2019a, b, c), and these peaks were observed on Sn-GP, indicating that there some changes in the peak position of S  =  O coordinated with Sn. According to the XRD spectrum (Additional file 1: Fig. S1B), a strong diffraction peak appeared at around 2*θ* of 26.5 corresponding to the carbon (002) diffraction peak of GP. Sn-GP still had a GP specific diffraction peak at 26.5° in the crystal plane and maintained the structure of GP without the peaks of tin compounds, verifying that the Sn-GP synthesis performance didn’t affect significantly main structures of GP (Truszkiewicz et al. 2020). According to the above results, Sn-GP and GP did not differ significantly in structure, and GP and tin exhibited very good biocompatibility. Sn-GP was a porous heterogeneous catalyst, which favored the transformation of biomass into FAL.

### Co-catalysis by combined Sn-GP and organic acids

**Fig. 2:**
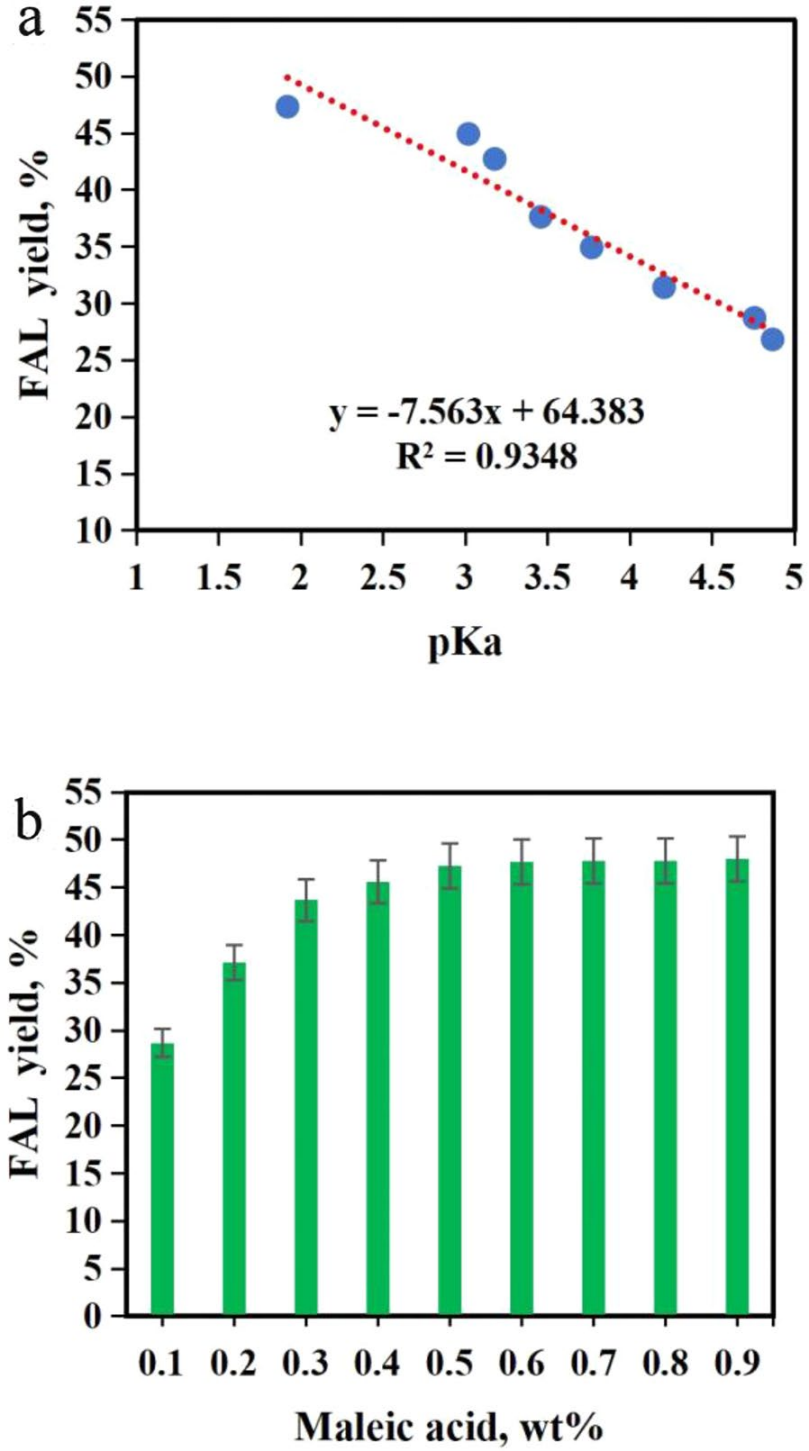
Effect of organic acid with different pKa value on the yield of FAL in the acidic condition (Corncob 3.0 g, Sn-GP 3.6 wt%, 170 °C, 0.5 h, water 40 mL) (**a**); Effect of maleic acid dose (0.1–0.9 wt%) on the yield of FAL (Corncob 3.0 g, Sn-GP 3.6 wt%, 170 °C, 0.5 h, water 40 mL) (**b**)

Organic acids could enhance the FAL formation (Choudhary et al. 2011). Several organic acids including fumaric acid (pKa  = 3.02), oxalic acid (pKa  = 1.25), maleic acid (pKa  = 1.92), glyoxalic acid (pKa  = 3.18), malic acid (pKa  = 3.46), citric acid (pKa  = 3.13), formic acid (pKa  = 3.77), succinic acid (pKa  = 4.21), acetic acid (pKa  = 4.76), and propionic acid (pKa  = 4.87) (0.5 wt%) were used to assist Sn-GP for co-catalysis of CC into FAL. FAL yields and pKa values of organic acid in the co-catalytic system were fitted in a linear equation FAL yield  = − 7.563 ×  pKa  +  64.383 (*R*^2^  = 0.9348) (Fig. 2a). As the pKa values increased from 1.92 to 4.87, the FAL yields decreased from 47.3% to 26.8%. Probably, the pKa values increased, the rates of decomposition of acidic protons became slower and their number decreased, which reduced the dehydration reaction and led to low FAL yields. Among these organic acids, maleic acid (pKa  = 1.92) gave the highest FAL yield (47.3%), indicating that maleic acid could assist solid acids to effectively promote FAL synthesis from biomass (Enslow and Bell 2015). Co-catalysis with Sn-GP and organic acids both enhanced CC conversion into FAL with higher yields compared to catalysis by Sn-GP without organic acid addition. Highest FAL yield (47.3%, based on CC) was achieved by co-catalysis with maleic acid and Sn-GP, which was twofolds of that (25.7%) with Sn-GP only. Low pKa of maleic acid might easily dissociate H^+^, which would favor the *D-*xylose dehydration into FAL (Janis et al. 2001). Using maleic acid (0.5 wt%) as catalyst in the absence of Sn-GP, the furfural yield was achieved at 6.7%. To further improve the generation of FAL by Sn-GP (3.6 wt%), the dosage of maleic acid (0.1–0.9 wt%) was evaluated on the FAL yields (Fig. 2b). FAL yield gradually increased from 28.7 to 47.3% when maleic acid dose was raised from 0.1 to 0.5 wt%. Further increasing its dose from 0.5 to 0.9 wt%, FAL yields had no significant change. Therefore, the appropriate maleic acid dose was found at 0.5 wt%.

### Optimization of converting biomass to FAL via co-catalysis with Sn-GP and maleic acid

**Fig. 3:**
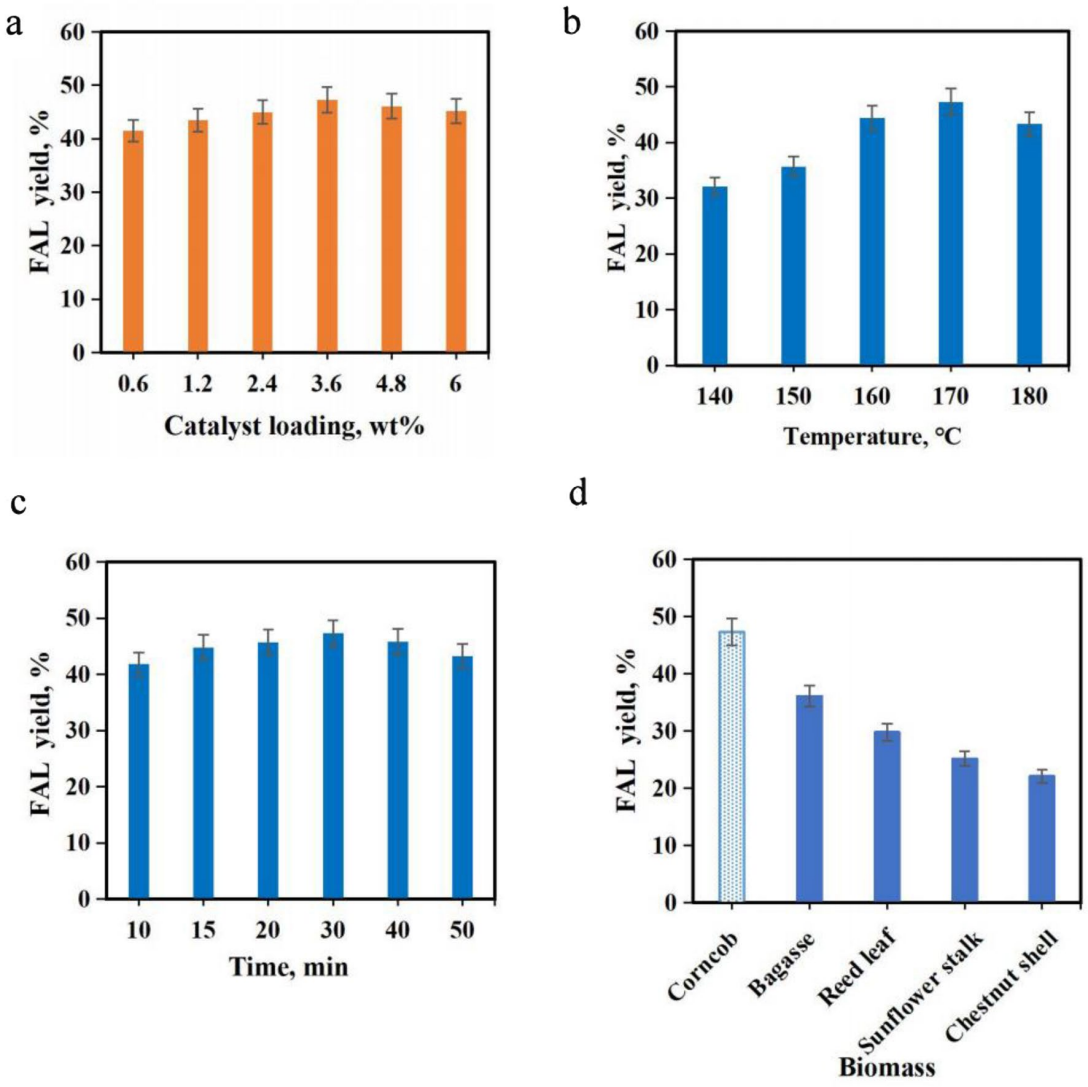
Effects of the Sn-GP dose (0.6–6.0 wt%) on FAL yields (Corncob 3.0 g, maleic acid 0.5 wt%, 170 °C, 0.5 h, water 40 mL) (**a**); Effects of reaction temperature (140–180 °C) on FAL yields (Corncob 3.0 g, Sn-GP 3.6 wt%, maleic acid 0.5 wt%, 0.5 h, water 40 mL) (**b**); Effects of reaction time (10–50 min) on FAL yields (Corncob 3.0 g, Sn-GP 3.6 wt%, maleic acid 0.5 wt%, 170 °C, water 40 mL) (**c**); Effects biomass types on FAL yields (Biomass 3.0 g, Sn-GP 3.6 wt%, maleic acid 0.5 wt%, 0.5 h, 170 °C, 0.5 h, water 40 mL) (**d**)

To further enhance Sn-GP’s catalytic efficiency, four factors including Sn-GP dosage, catalytic temperature, types of biomass and catalytic time were examined on the effects of FAL generation in the presence of maleic acid (0.5 wt%) (Fig. 3). FAL yields were raised as the Sn-GP dose increased from 0.6 to 3.6 wt%. At 3.6 wt%, FAL yield reached 47.3%. In the range of 3.6–6.0 wt%, FAL yields dropped slightly (Fig. 3a). The optimal Sn-GP dosage was 3.6 wt%. The catalytic temperature played an important role in converting biomass into yield FAL (Fig. 3b). At 140 °C, low yield of FAL arrived at 32.1%. With the increase of temperature, FAL yield increased, and the maximum FAL yield reached 47.3% at 170 °C. Over this temperature, the FAL yield started to decrease slightly. Probably, lower catalytic temperature couldn’t provide enough energy for catalyzing biomass, while higher performance temperature favored the generation of FAL molecules and promoted emergency of undesired side-reactions (Li et al. 2021a, b). The performance time had a profound influence on the FAL yields (Umapathi et al. 2020). When the catalytic time was below 30 min, FAL yields gradually increased with the prolonged reaction time (Fig. 3c). Over 30 min, FAL yields began to dropped slightly. Long performance time would result in FAL degradation and led to a decreased FAL yield.

To test the catalytic ability of co-catalysts, CC (31.4%), sugarcane bagasse (xylan 25.0%), reed leave (xylan 21.1%), sunflower straw (xylan 19.1%) and chestnut shell (xylan 18.2%) were used as feedstocks to produce FAL. Figure 3d demonstrates the effect of using different biomass as substrate on FAL yield. Highest FAL yield arrived at 47.3% when CC was used as substrate. However, lower FAL yields (22.1–36.1%) were obtained using sugarcane bagasse, reed leave, sunflower straw and chestnut shell as feedstocks. This might be due to the higher hemicellulose content of biomass and the better conversion of hemicellulose to xylose in the aqueous phase (Lee et al. 2019).

**Fig. 4:**
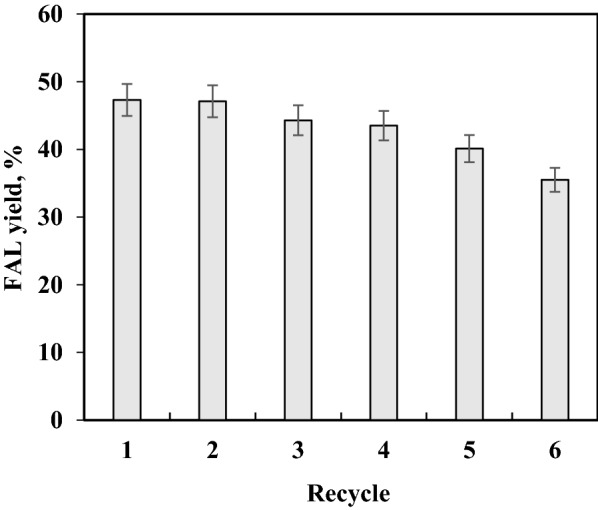
Reuse of Sn-GP

To evaluate the stability of Sn-GP, its reusability was investigated via co-catalysis of CC with maleic acid (0.5 wt%) and Sn-GP (3.6 wt%) in water. Reuse of Sn-GP was conducted for six runs (Fig. 4). In 1st batch, FAL yield reached 47.3%. After 2th batch, FAL yields declined gradually. From 1st to 5th run, FAL yields were over 40%. At 6th run, FAL yield dropped to 35.5%. Clearly, Sn-GP had high stability for transforming CC into FAL.

To sum up, FAL yield arrived at 47.3% from CC (75 g/L) by cocatalysis with 0.5 wt% of maleic acid and 3.6 wt% of Sn-GP at 170 °C in 30 min. FAL liquor was consisted of 83.2 mM FAL, 5.3 mM HMF, 2.1 mM HCOOH, and 1.2 mM levulinic acid. Recently, various tin-based solid acid catalysts were used for transforming biomass or *D*-xylose into FAL (Table 1). Using Sn-adamellite or Sn-sepiolite as catalyst, biomass was required to be treated with alkalic solution prior to transformation into FAL in the acidic reaction media, and 42.2–57.5% of FAL yield were obtained (Peng et al. 2019; Yang et al. 2020), and excessive alkaline and acid were used. Using *D*-xylose as substrate, co-catalysis with oxalic acid (0.35 wt%) and SO_4_^2−^/SnO_2_-argil (3.6 wt%) could give FAL (107.6 mM) at 50.8% yield (Xue et al. 2018). After corncob was hydrolyzed with oxalic acid (0.5 wt%) at 140 °C in 40 min, the obtained *D*-xylose could be catalyzed into FAL (67.3 mM) at 74.3% yield with SO_4_^2−^/SnO_2_-kaoline (3.5 wt%) in toluene–water (1:2, v:v) containing 10 mM OP-10 at 170 °C within 30 min (He et al. 2017b). It is known that toluene is highly toxic, and it should be avoided to be used. Co-catalysis of HCOOH (1.0 wt%) and SO_4_^2−^/SnO_2_-MMT (2.0 wt%) was used to catalyze corncob into FAL (77.8 mM) in the yield of 40.2% at 180 °C within 10 min under microwave (600 W) (Huang et al. 2019). However, high energy consumption was required. Sn-BTN (3.5 wt%) transformed corncob into FAL (103.4 mM) at 53.3% yield in MIBK–water (5:5, v:v; pH 1.0) at 170 °C within 30 min (Zhang et al. 2020c). Excessive organic solvent MIBK was used. In our study, corncob was converted into FAL (83.2 mM) at 47.3% yield by Sn-GP (3.6 wt%) and maleic acid (0.5 wt%) in water at 170 °C within 30 min. Although the FAL yield was not high, organic solvent was avoided, and the FAL preparation process from biomass was easier to perform compared to previous reporsts (Huang et al. 2019; Peng et al. 2019; Yang et al. 2020).

**Table 1:** Synthesis of furfural from *D*-xylose or biomass by tin-based solid acids

Reactant	Catalyst	Reaction conditions	FAL yield (FAL Con.)	Reaction media	Ref.
Bamboo shoot shell (BSS)	Sn–adamellite	170 °C, 30 min	57.5% (70.2 mM)	H_2_O, pH 1.0	Yang et al. (2020)
Alkali-treated dewaxed rice straw	Sn–sepiolite	170 °C, 20 min	42.2% (97.8 mM)	H_2_O, pH 1.0	Xue et al. (2018)
*D*-Xylose	SO_4_^2−^/SnO_2_-argil	180 °C, 20 min	50.8% (107.6 mM)	H_2_O, oxalic acid (0.35 wt%)	He et al. (2017b)
Corncob-derived *D*-xylose	SO_4_^2−^/SnO_2_–kaoline	170 °C, 30 min	74.3% (67.3 mM)	H_2_O–toluene (1:2, v:v), OP-10 10 mM	Huang et al. (2019)
Corncob	SO_4_^2−^/SnO_2_-MMT	180 °C, 10 min	40.2% (77.8 mM)	H_2_O–HCOOH (1.0 wt%), Microwave (600 W)	Zhang et al. (2020c)
Corncob	Sn-BTN	170 °C, 30 min	53.3% (103.4 mM)	MIBK-H_2_O (1:1), pH 1.0	Peng et al. (2019)
Corncob	Sn-GP	170 °C, 30 min	47.3% (83.2 mM)	H_2_O, maleic acid (0.5 wt%)	This study

### Chemoenzymatic cascade catalysis of CC into FOL and FA

FOL production from FAL catalyzed by 0.6% Pt0.4Sn/SiO_2_ catalyst at 100 °C and 20 bar H_2_ pressure was achieved in 47% yield (Gong et al. 2017). The Zr-PW catalyst catalyzed the reaction of FAL at 120 °C for 60 min, achieving FOL yield at 98.6% (Xu et al. 2019). The PhP-Hf (1:1.5) catalyst converted FAL to FOL in 2-propanol medium with a 97.6% conversion at 120 °C for 2 h (Li et al. 2019a, b, c). Industrially, FA was produced by the Cannizzaro reaction between FAL and aqueous sodium hydroxide (NaOH), accompanied by FOL (Shi et al. 2019). Biocatalysis has emerged as an attractive route for producing important in high yields and selectivity under mild performance conditions.

**Fig. 5:**
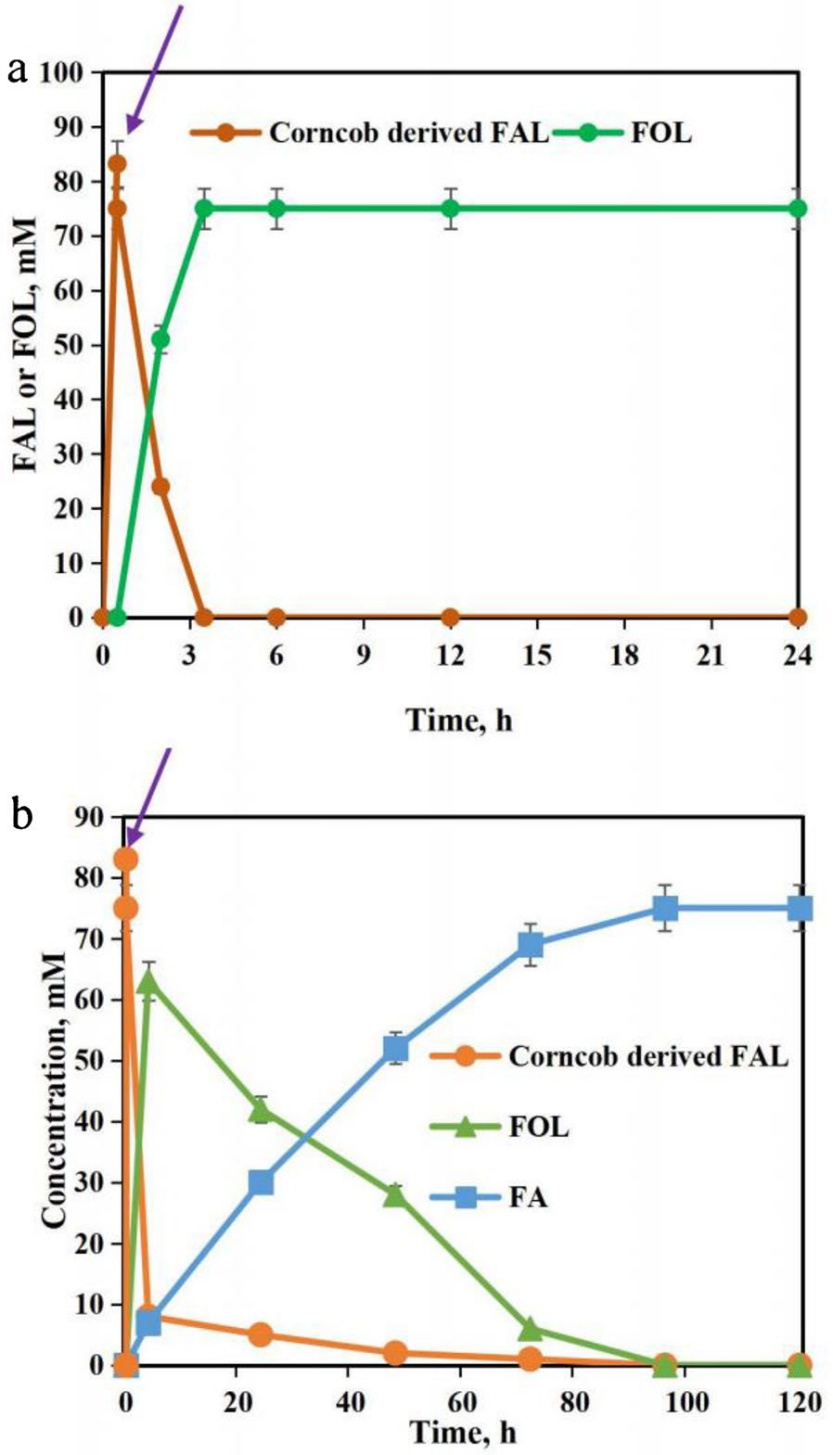
Reaction process for one-pot production of FOL (**a**) and FA (**b**) from corn cobs catalyzed by chemoenzymatic cascade catalysis with maleic acid-assisted Sn-GP chemocatalyst and whole-cell biocatalyst. (Arrow: pH adjustment)

To efficiently biotransform FAL into FOL, cells of *E. coli* CG-19, *E. coli* CCZU-K14 and *E. coli* CCZU-A13 were used as reductase biocatalysts for FAL-reducing reaction. FOL was prepared by bioreduction reaction of CC-derived FAL using CG-19 (35 °C, pH 7.5), CCZU-K14 (30 °C, pH 6.5) and CCZU-A13 (30 °C, pH 6.5), respectively. All three whole-cell catalysts were able to completely convert 75.0 mM FAL into FOL (Table 2), CCZU-A13, CCZU-K14, and CG-19 required 12, 8, and 3 h for completely transforming FAL to FOL, respectively. Among these three whole-cell catalysts, CG-19 had the highest catalytic efficiency, which could effectively transform CC-derived FAL (Fig. 5a). In an aqueous phase system (170 °C), CC was catalyzed to yield 83.2 mM FAL by co-catalyst with maleic acid and Sn-GP in 0.5 h. The pH of the liquid was then adjusted to 7.5, and glucose (glucose-to-FAL molar ratio 1.5:1) and *E. coli* CG-19 (50 g/L) were added to FAL liquor, and the dilute FAL liquor containing 75.0 mM FAL. FAL was completely bioreduced for 3 h at 35 °C. The reaction was completed and the FOL yield reached 100%. When *E. coli* CG-19 whole cells catalyzed CC conversion to FOL, no significant inhibition was observed. Biobased FOL was prepared from CC by a designed chemoenzymatic cascade catalysis with Sn-GP and CG-19 cells. In a previous report, 20 mM FAL was transformed to FOL in 71% yield by UiO-66 (75 mg) in 25 mL of organic amine at 170 °C in 10 h (Qiu et al. 2020). FAL (10 mM) was completely converted to FOL at 24 h catalyzed by *M. deltae* growth cells (Belay et al. 1997). 30 mM of FAL was converted to FOL within 24 h using wild strain of *B. cereus* with a conversion rate of 80% (Rodriguez et al. 2021). Clearly, the catalytic production of FAL using *E. coli* CG-19 not only had higher FAL tolerance, but also had higher catalytic yield and selectivity.

**Table 2:** Bioreduction of FAL to FOL with reductase-producing strains

Strains	Reaction conditons	Initial FAL, mM	FOL yield, %
*E. coli* CG-19	3 h, 35 °C, pH 7.5	75.0	100
*E. coli* CCZU-K14	8 h, 30 °C, pH 6.5	75.0	100
*E. coli* CCZU-A13	12 h, 30 °C, pH 6.5	75.0	100

Whole-cell catalysts are essential in the biological oxidation of FAL to FA (Yang et al. 2020; Zhu et al. 2020a, b). *E. coli* TS, *E. coli* HMFOMUT, and *E. coli* BH were selected as FAL-oxidizing whole-cell catalysts under their optimum reaction conditions. *E. coli* TS was able to completely convert CC-derived FAL (75.0 mM) to FA in 96 h (30 °C pH 7.0) (Table 3). HMFOMUT gave 80.2% FA yield in 120 h (30 °C pH 6.5). *E. coli* BH gave a low FA yield (60.3%) in 200 h (35 °C pH 7.5). Clearly, *E. coli* TS whole cell was the best candidate. Dilute CC-derived FAL (75.0 mM) was transformed with TS cells. Within 4 h, the FOL formation rate was faster than FA. At 4 h, the FOL concentration was obtained at the highest value (63.0 mM), and FA was obtained at 7.1 mM. After 4 h, FOL was oxidized into FA. After 96 h of biotransformation, FAL was completely oxidized to FA (Fig. 5b). No inhibitory effect was observed when *E. coli* TS whole cells were used to catalyze the conversion of CC-derived FAL to FA. The production of FA from sustainable and inexpensive CC using an effective hybrid strategy of tandem catalysis with Sn-GP catalysts and *E. coli* TS biocatalysts is an economical approach. In the previous report, FA was formed by Cannizzaro reaction between FAL and aqueous NaOH solution accompanied by the formation of FOL (Douthwaite et al. 2017). Dehydrogenase (SAPDH) from *C. testosteroni* was used to transform 100 mM of FAL to FA with the conversion of 95–98% in 96 h (Shi et al. 2019). In this study, *E. coli* TS could completely transform CC-derived FAL into FA with high catalytic efficiency and selectivity in 90 h.

**Table 3:** Biological oxidation of FAL to FA with dehydrogenase-producing strains

Strains	Reaction conditons	Initial FAL, mM	FA yield, %
*E. coli* TS	96 h, 30 °C, pH 7.0	75.0	100
*E. coli* HMFOMUT	120 h, 30 °C, pH 6.5	75.0	80.2
*E. coli* BH	200 h, 35 °C, pH 7.5	75.0	60.3

**Fig. 6:**
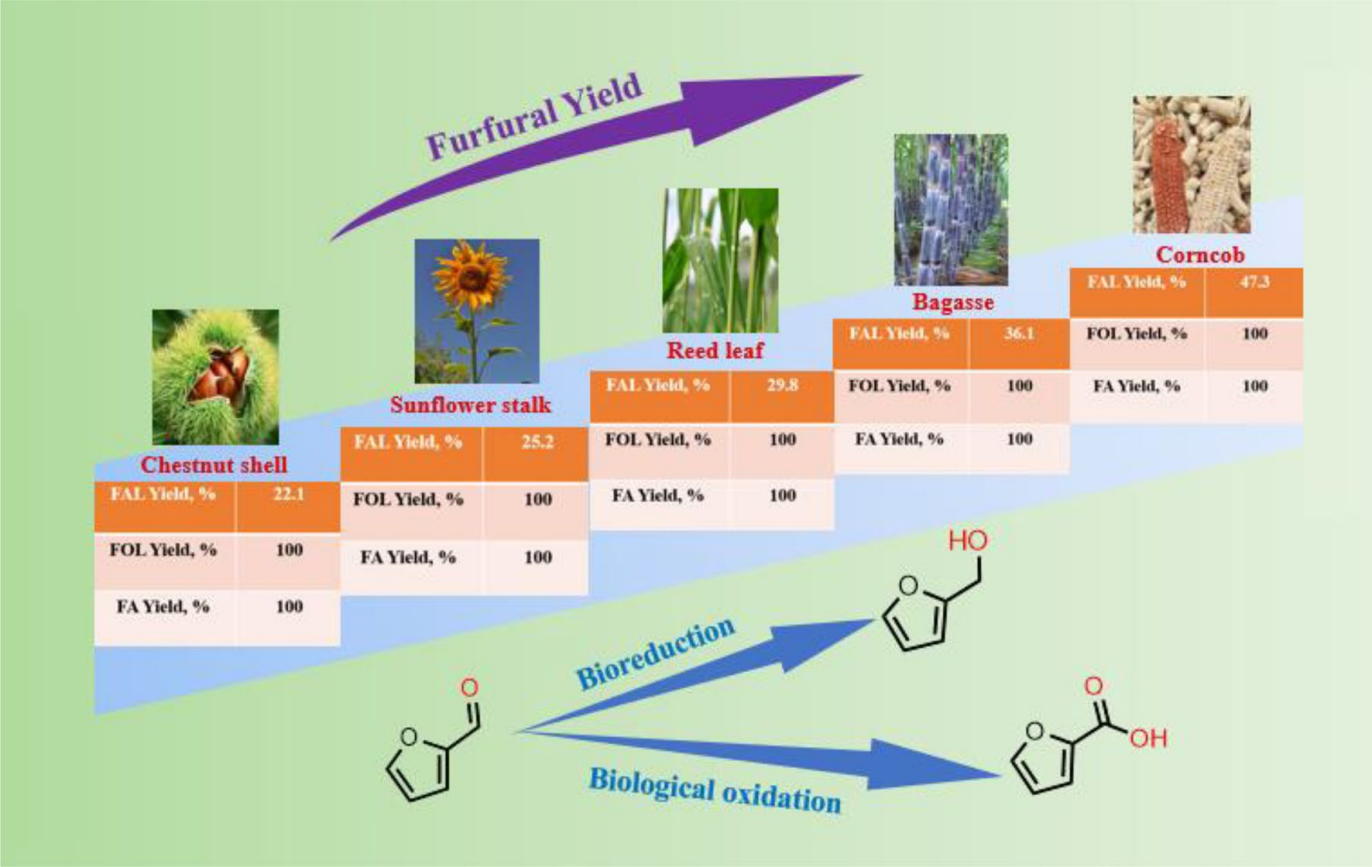
Tandem valorization of corncob, sunflower stalk, chestnut shell, reed leaf, and sugarcane bagasse into FA and FOL

To explore the catalytic ability of US-Sn-CNS, five kinds of biomasses, including corncob (CC), sunflower stalk (SFS), chestnut shell (CNS) reed leaf (RL), and sugarcane bagasse (SCB), were used as feedstocks for the synthesis of FAL at 170 °C in 0.5 h. The FAL yields were obtained as follows: *Y*_(CC)_  = 47.3%  >  *Y*_(SCB)_  = 36.1%  >  *Y*_(RL)_  =  29.8%  >  *Y*_(SFS)_  =  25.2%  >  *Y*_(CNS)_  =  22.1%. Furthermore, these obtained FAL liquors could be completely biotansformed into FA and FOL with high yields (100% analytical yields, based on FAL) (Fig. 6), respectively. Development of sustainable process for production of bio-based furans from biomass has gained great interest recently. Cost-efficient production of FA and FOL from waste biomass in benign reaction media is under progress.

### Mass balance form biomass to furans by chemoenzymatic synthesis

**Fig. 7:**
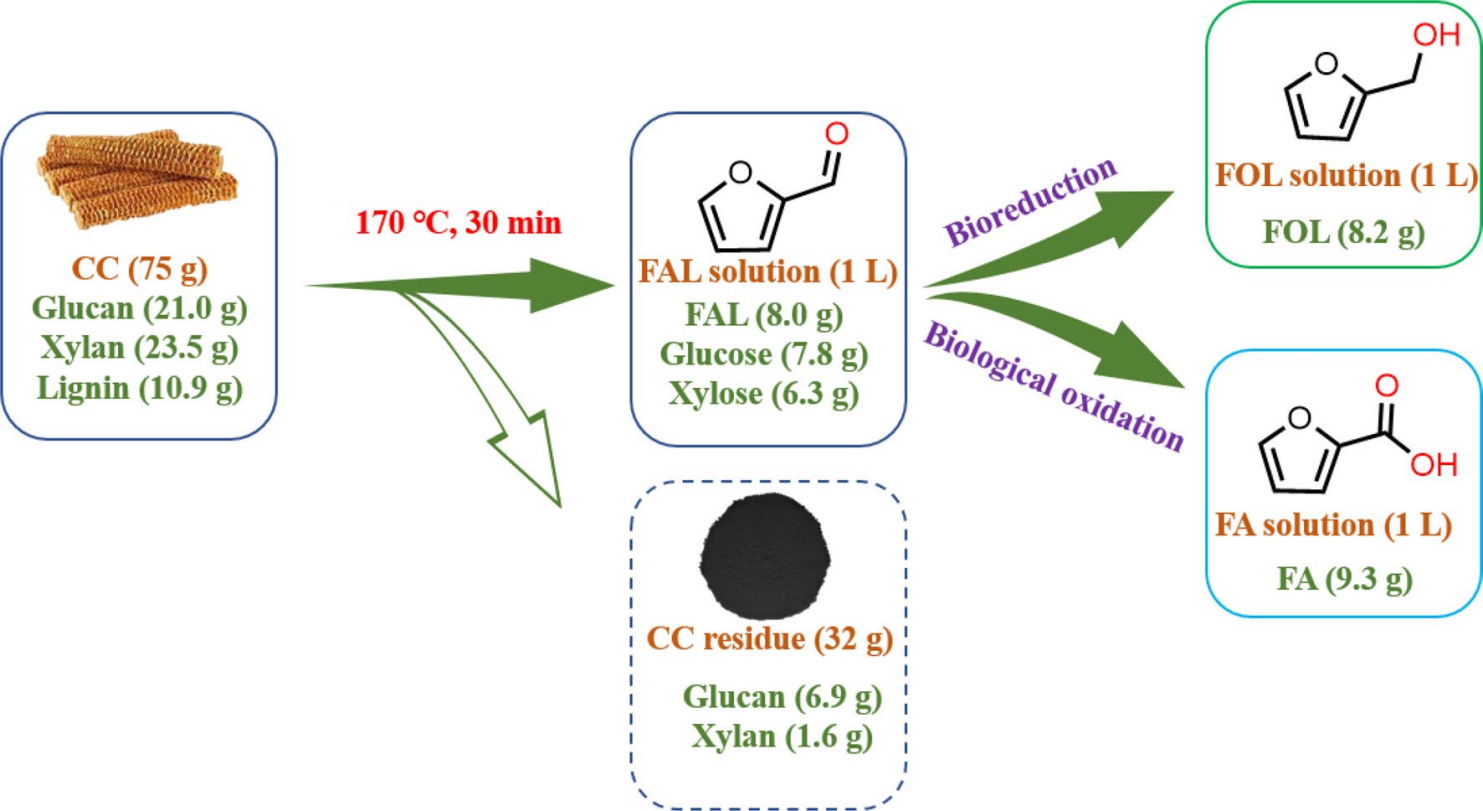
Mass balance from biomass into furran chemicals

The mass balance of this cascade reaction was depicted involving CC components, Sn-GP mediated CC catalysis to FAL and bioconversion of FAL to furans (FOL and FA) (Fig. 7). In a 2.5-L autoclave containing 1 L of water, CC (75.0 g) consisting of 21.0 g glucan, 23.5 g xylan and 10.9 g lignin was chemically catalyzed by Sn-GP at 170 °C in 30 min to give 1.0 L FAL solution consisting of 6.3 g *D*-xylose, 7.8 g glucose, 8.0 g FAL under stirring at 500 rpm. Subsequently, CC-derived FAL (8.0 g) was biologically transformed to 8.2 g FOL using *E. coli* CG-19 (50.0 g, wet weight) over 3 h at pH 7.5 and 35 °C. Yield of 0.109 g FOL/g CC (0.35 g FOL/g xylan in CC) was achieved. In addition, CC-derived FAL (8.0 g) was transformed to 9.3 g FA using *E. coli* TS (50.0 g, wet weight) at 30 °C and pH 7.0 for 96 h. A yield of 0.124 g FA/g CC (0.40 g FA/g xylan in CC) was obtained.

Clean production of bio-based furans from available renewable resource is crucial to achieve sustainable biorefinery process (Bu et al. 2020; Cheng et al. 2020; Di et al. 2021; Hao et al. 2021; Zhang et al. 2020a). Chemoenzymatic cascade transformation of biomass into FOL and FA with high yield is of great interest via tandem reaction with chemocatalysis and biocatalysis. In the aqueous system, Sn-GP, which was prepared from GP as carrier, was utilized to produce FAL from CC. Organic acid-assisted solid acid could give higher yields of FAL. The CC-derived FAL could be biotransformed to FOL and FA by the reductase and dehydrogenase biocatalysts, respectively. One-pot chemoenzymatic cascade transformation was successfully developed for efficient valorisation of biomass to furans. This strategy could be conducted in a simple operation with high catalytic efficiency. In future, it would be an interesting topic to establish a greener chemical–biological pathway to improve FAL yields and thus further improve FOL and FA yields.

## Conclusions

In this study, organic acid-assisting GP-based heterogeneous catalysis was explored for transforming biomass into FAL. Co-catalysis of milled CC powder (7.5 wt%) into FAL at 47.3% yield in water containing maleic acid (0.5 wt%) and Sn-GP (3.6 wt%) at 170 °C for 0.5 h. The formed FAL liquor could be effectively transformed into furans (FOL and FA) via one-pot chemoenzymatic cascade catalysis. A sustainable strategy was constructed to valorize renewable lignocellulose biomass to value-added furan biobased chemicals in a tandem reaction with chemocatalyst and biocatalysts in an environmentally friendly manner.

### Supplementary Information

**Additional file 1: Figure S1. **FTIR image of GP and Sn-GP (**A**) and XRD images of GP and Sn-GP (**B**). **Table S1. **Surface and pore characteristics of GP and Sn-GP.

## Data Availability

All data supporting this article’s conclusion are available.

## Abbreviations

FAL: Furfural
FOL: Furfuryl alcohol
FA: Furoic acid
CC: Corncob
SFS: Sunflower stalk
CNS: Chestnut shell
SCB: Sugarcane bagasse
RL: Reed leaf
GP: Graphite
