# Supplementary material for: Valorisation of corncob into furfuryl alcohol and furoic acid via chemoenzymatic cascade catalysis
Source: Bioresour Bioprocess. 2021 Nov 16;8(1):113. doi: 10.1186/s40643-021-00466-3 (PMC10991097; doi:10.1186/s40643-021-00466-3)
Supplement: Supplementary file 1 — Additional file 1: Figure S1. FTIR image of GP and Sn-GP (A) and XRD images of GP and Sn-GP (B). Table S1. Surface and pore characteristics of GP and Sn-GP. [file 40643_2021_466_MOESM1_ESM.docx]

**Support Information**

**Figure Caption**

**Fig. S1.** FTIR image of GP and Sn-GP (A) and XRD images of GP and Sn-GP (B).

**Table Captions**

**Table. S1.** Surface and pore characteristics of GP and Sn-GP..


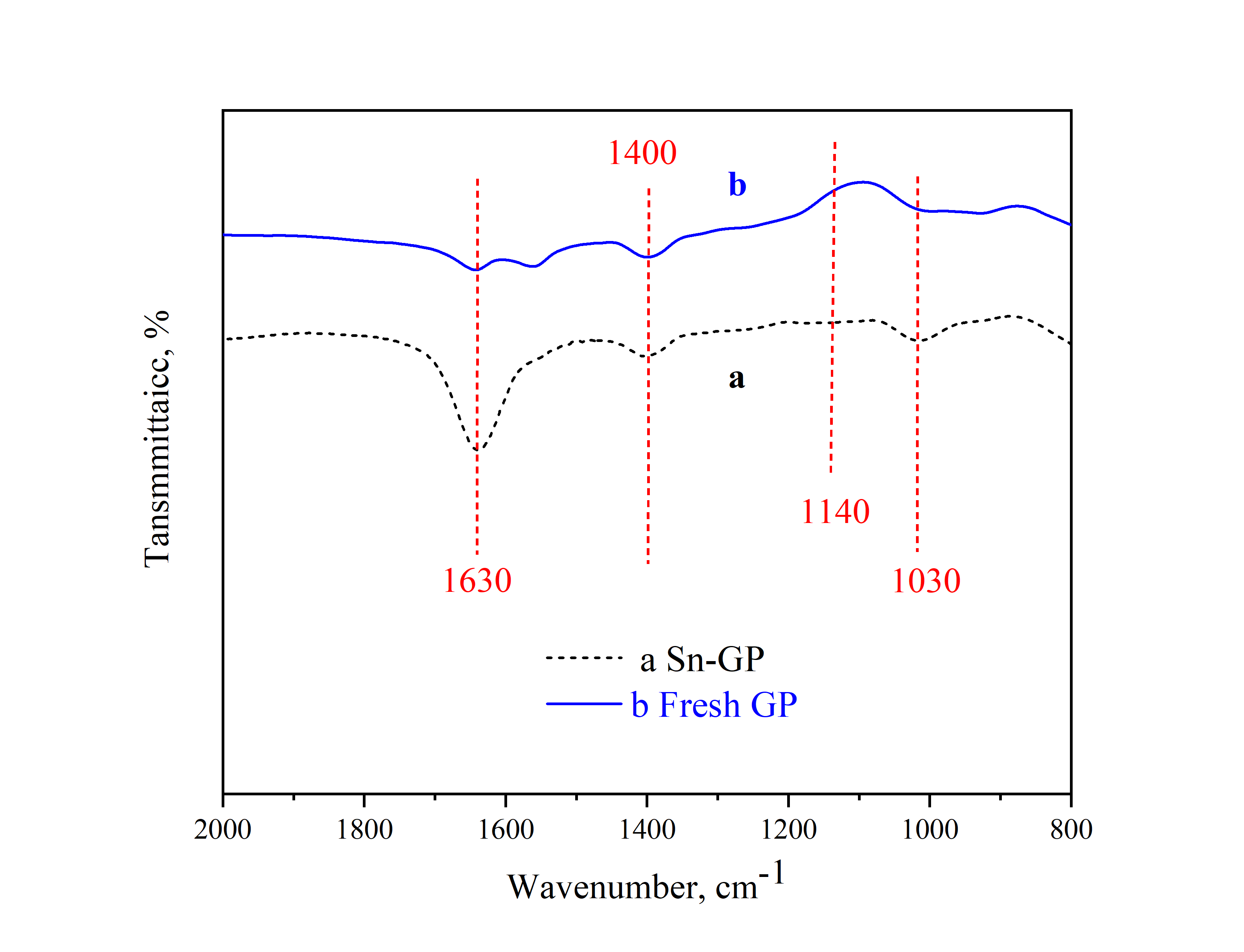

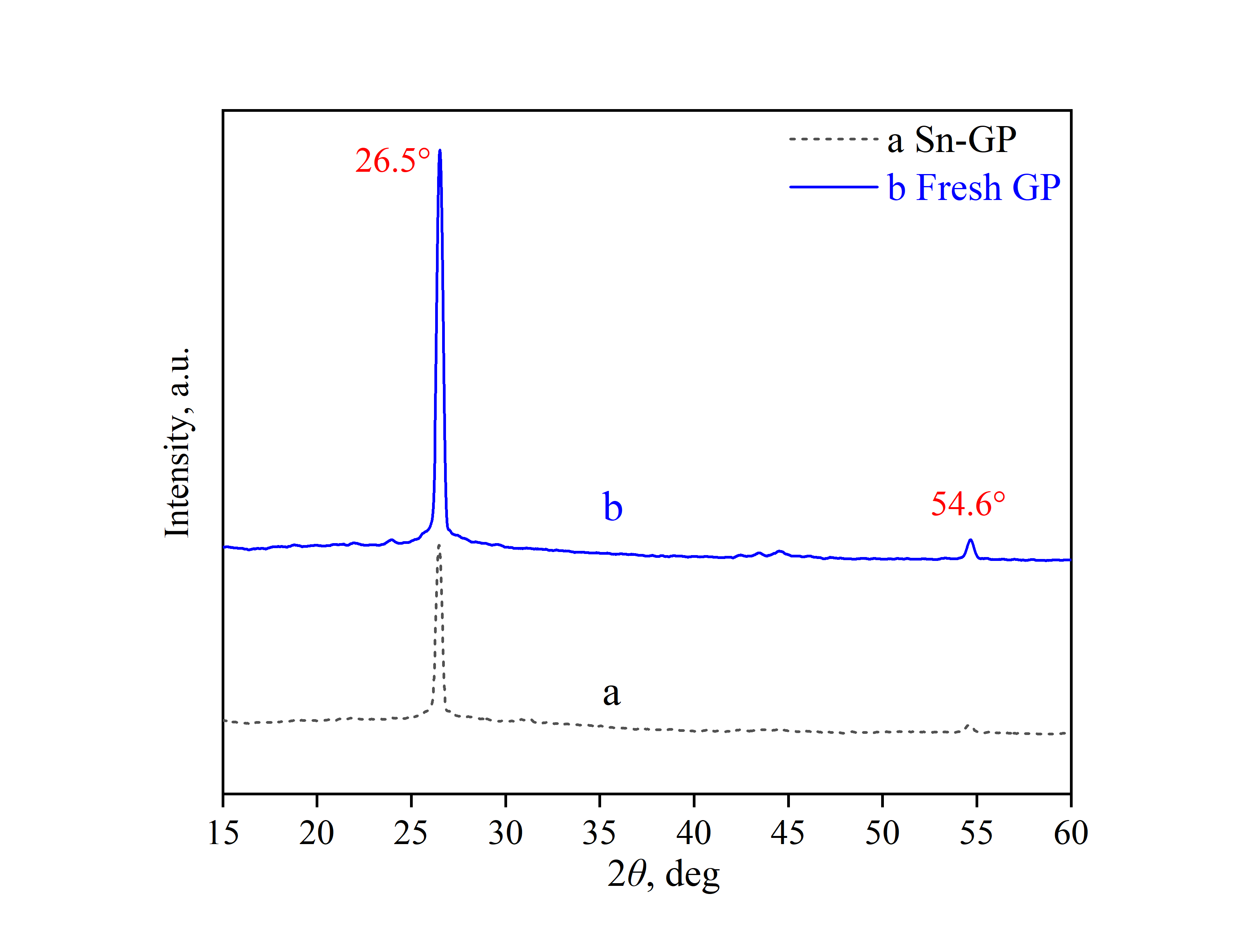


B

A

**Fig. S1.**

**Table. S1.** Surface and pore characteristics of GP and Sn-GP.

| **Solid sample** | **Specific surface area, m^2^/g** | **Pore volume,**  **cm^3^/g** | **Pore diameter,**  **nm** |
| --- | --- | --- | --- |
| GP | 0.5 | 0.01 | 135.2 |
| Sn-GP | 29.4 | 0.03 | 5.8 |
